# Safety and efficacy of peptide receptor radionuclide therapy in neuroendocrine tumors: A single center experience

**DOI:** 10.1371/journal.pone.0298824

**Published:** 2024-05-15

**Authors:** Vineeth Sukrithan, Heather Armbruster, Sherise Rogers, Sherry Mori Vogt, Cassandra Grenade, Claire Verschraegen, Ye Zhou, Ashima Goyal, Mona Natwa, Akram Hussein, Hallie Barr, Dramane Konate, Rochelle Batdorf, Andrew Brown, Bonnie Williams, Songzhu Zhao, Lai Wei, Menglin Xu, Manisha H. Shah, Bhavana Konda

**Affiliations:** 1 Division of Medical Oncology, Department of Internal Medicine, The Ohio State University and Arthur G. James Cancer Center, Columbus, Ohio, United States of America; 2 Department of Pharmacy, The Ohio State University, Columbus, Ohio, United States of America; 3 Division of Hematology Oncology, Department of Medicine, University of Florida, Gainesville, FL, United States of America; 4 Division of Pharmacy, Medical University of South Carolina, Charleston, South Carolina, United States of America; 5 Division of Hematology Oncology, Department of Medicine, Ohio Health, Delaware, OH, United States of America; 6 Department of Radiology, The Ohio State University, Columbus, Ohio, United States of America; 7 Department of Nuclear Medicine Pharmacy, The Ohio State University, Columbus, Ohio, United States of America; 8 Department of Environmental Health and Safety, The Ohio State University, Columbus, Ohio, United States of America; 9 Cardinal Health, Dublin, Ohio, United States of America; 10 Department of Biostatistics, The Ohio State University, Columbus, Ohio, United States of America; Emory University, UNITED STATES

## Abstract

Peptide receptor radionucleotide therapy (PRRT) with ^177^Lu-dotatate is widely used for the treatment of patients with neuroendocrine tumors (NETs). We analyzed data from 104 patients with NETs treated with ^177^Lu -dotatate at a US academic center between December 2017 and October 2020 to better understand patterns of long-term efficacy, safety, and toxicity in the real-world setting. ^177^Lu-dotatate (200 mCi) was administered every eight weeks for four doses. The most common sites of primary disease were small intestine NETs (n = 49, 47%), pancreatic NETs (n = 32, 31%), and lung NETs (n = 7, 7%). Twenty-seven percent had Ki-67 <3%, 49% had Ki-67 between 3–20%, and 13.5% had Ki-67 >20%. The cohort had been pretreated with a median of two prior lines of treatment. Forty percent had received prior liver-directed treatment. Seventy-four percent of patients completed all four doses of treatment. The objective response rate was 18%. The median time-to-treatment failure/death was significantly longer for small-bowel NETs when compared to pancreatic NETs (37.3 months vs. 13.2 months, p = 0.001). In a multivariate model, Ki-67, primary site, and liver tumor burden ≥50% were found to independently predict time-to-treatment failure/death. Around 40% of patients experienced adverse events of ≥grade 3 severity. Treatment-related adverse events leading to discontinuation of therapy happened in 10% of patients. Preexisting mesenteric/peritoneal disease was present in 33 patients; seven of these patients developed bowel-related toxicities including two grade 5 events. We also report two cases of delayed-onset minimal change nephrotic syndrome, which occurred 14 and 27 months after the last dose of PRRT. Lastly, we describe six patients who developed rapid tumor progression in the liver leading to terminal liver failure within 7.3 months from the start of PRRT, and identify potential risk factors associated with this occurrence, which will need further study.

## Introduction

Radiolabeled somatostatin analogs belong to a class of therapies called peptide receptor radionuclide therapy (PRRT), which have shown utility in the treatment of somatostatin-receptor (SSTR)-expressing neuroendocrine tumors (NET). The β-emitters ^90^Y and ^177^Lu have shown the most evidence of safety and efficacy of the radio-ligands studied. Lu-177 dotatate, the radionuclide ^177^Lutetium (^177^Lu) linked to a somatostatin analog was approved by the Food and Drug Administration (FDA) in January 2018 for the treatment of metastatic or advanced somatostatin receptor-positive gastroenteropancreatic neuroendocrine tumors (GEP-NETs) based on the results of the multinational randomized controlled phase 3 NETTER-1 study (NCT01578239) [1]. The trial enrolled patients with well-differentiated mid-gut neuroendocrine tumors (Ki-67 ≤ 20%) who had progressed on prior treatment with octreotide long-acting repeatable (LAR); they were randomized to a control arm treated with high-dose octreotide LAR and a treatment arm consisting of ^177^Lu‐dotatate in combination with high-dose octreotide LAR. Treatment with ^177^Lu‐dotatate was associated with an objective response rate of 18% and a progression-free survival rate at 20 months of 65.2% in the treatment group, compared to 10.8% in the control arm. Median overall survival was 48.0 months in the ^177^Lu-dotatate arm and 36.3 months in the control arm (HR = 0.84, p = 0.30) [2].

We aimed to collect and analyze real-world experience from patients treated with PRRT, many of whom are heavily pretreated and have multiple co-morbidities. Prior efforts have identified differential efficacy based on site of primary disease in mixed NET populations which needs confirmation [3–5]. Our study also specifically focused on studying and reporting rare but important under-appreciated adverse events such as nephrotic syndrome, bowel toxicities, and accelerated progression [6–9].

## Methods

In this retrospective study, we reviewed medical records of all patients who began treatment with PRRT with ^177^Lu-dotatate between December 2017 and October 2020 at The Ohio State University for the treatment of neuroendocrine tumors. Written approval was obtained from the Institutional Review Board of The Ohio State University (2018C0187). As all research involved materials (data, documents, and records) that were collected solely for non-research purposes (i.e., medical treatment or diagnosis) and therefore represented only minimal-risk research, permission was obtained for a waiver of consent. Data was accessed for research purposes from January 1, 2019 through January 31, 2023.

Eligibility criteria: Patients were considered eligible for treatment if they met criteria for adequate end-organ function at baseline as defined as: WBC >2,000, platelets >75,000, Hgb >8.0 g/dL, creatinine clearance (CrCl) >45 mL/min, or serum creatinine <1.7 mg/dL. If serum creatinine was >1.7, treatment was permissible if there was ≤40% increase in serum creatinine/CrCl from baseline. In terms of liver function, total bilirubin had to be less than three times the upper limit of normal (ULN), AST/ALT less than five times ULN, and serum albumin >3.0 g/dL. If serum albumin was <3 g/dL, treatment was still permissible if the prothrombin ratio was <70%.

Treatment: ^177^Lu-dotatate was administered in the hospital-based infusion center in an outpatient setting every 8 weeks for 4 doses. Arginine-lysine amino acids (AA) 25gm/25gm in 1L normal saline was infused over 4 hours, and intravenous palonosetron 0.25mg was administered as a prophylactic anti-emetic starting 30 minutes before starting the AA infusion. ^177^Lu-dotatate was administered intravenously at a dose of 200 mCi over 20 to 30 minutes, using a dedicated peripheral IV line after 30 minutes of starting the AA infusion. Somatostatin analogs were not administered for at least 4 weeks prior to each ^177^Lu-dotatate dose. In patients with a need for symptom control, somatostatin analogs (SSAs) were administered >24 hours after the ^177^Lu-dotatate dose.

### Clinical, adverse event, and response assessments

Patients who received ≥2 PRRT doses had baseline anatomical imaging within 8 weeks of first dose and had anatomical imaging following ≥2 doses were considered evaluable for efficacy. Outpatient clinical evaluation including complete blood count with differential, comprehensive metabolic panel, and lactate dehydrogenase (LDH) occurred 2 weeks before and after each dose, and radiographic assessment using contrast CT/MRI was performed following 2 and 4 doses. Patients who received ≥1 PRRT dose(s) were considered evaluable for toxicity. Response assessment was done using the RECIST v1.1 criteria. Adverse effects were evaluated using CTCAE v5. Liver, mesenteric, and peritoneal tumor burden were independently visually estimated semi-quantitatively by two neuroendocrine tumor (NET) medical oncologists and categorized as present/absent (mesenteric/peritoneal disease) or <50% or ≥50% (liver tumor burden). Visual estimation of the liver tumor burden has good reproducibility, independent of experience, and has a high degree of inter-observer agreement between radiologists and non-radiologists [10, 11].

For survival data, time to treatment failure was defined as time from first dose of ^177^Lu-dotatate and date of next line of treatment (not including somatostatin analogs) or death.

### Statistical analysis

Descriptive statistics were used to summarize demographic and clinical information of patients. Discrete variables were described by frequencies and percentages, continuous variables were described by median and range, and time-to-event data were summarized by the Kaplan-Meier method to produce median survival time and its 95% CI. Group comparisons on survival time were conducted via log-rank tests. To investigate the factors contributing to the survival outcome, univariate Cox regression models were fit for each potential predictor (i.e., primary site, grade of disease, prior liver directed therapy, and liver tumor burden >50%). Those variables that showed a significant effect in the univariate model served as the joint predictors in the multivariate Cox regression model. All the tests used an alpha of 0.05.

## Results

A total of 104 patients were identified who had been treated with at least one dose of PRRT, constituting the total population assessable for adverse events. Patient characteristics (Table 1) included 55% males and 45% females. The racial makeup of the cohort was 90% non-Hispanic white. The median age was 65 years (range 30–84). All patients had metastatic NETs. The three most common primary malignancies were small intestine NETs (n = 49, 47%), pancreatic NETs (n = 32, 31%), and lung NETs (n = 7, 7%). The rest of the patients had hindgut NETs, pheochromocytoma/paraganglioma, gastric NETs, medullary thyroid cancer, and prostate NETs. Twenty-seven percent had Ki-67 <3%, 49% had Ki-67 between 3–20%, and 13.5% had Ki-67 >20%. For patients with Ki-67 >20%, the median Ki-67 was 30% (Range 20%-70%). Eleven percent had unknown Ki-67 percentages (Table 1). The cohort had received a median of two prior lines of treatment. Forty percent of patients had received three or more prior lines of therapy including SSA (80%), everolimus (30%), and capecitabine/temozolomide (40%).

**Table 1:** Patient characteristics.

Characteristics	N = 104 (100%)
**Age, median**	65
Range (years)	30–84
**Sex, No. (%)**	
Male	57 (55)
Female	47 (45)
**Race/Ethnicity, No. (%)**	
Non-Hispanic White Only	93 (90)
Black	3 (3)
Hispanic White	3 (3)
Mixed	2 (2)
Asian	1 (1)
Unknown	2 (2)
**Primary site for NET, No. (%)**	
Small intestine	49 (47)
Pancreas	32 (31)
Hindgut	5 (5)
Lung	7 (7)
Pheochromocytoma/Paraganglioma	5(5)
Others	6 (6)
**Liver tumor burden, No. (%)**	
<50%	84 (81)
≥50%	20 (19)
**Mesenteric/Peritoneal Disease**	
Yes	33 (32)
No	71 (68)
**Ki-67%, No. (%)**	
<3%	28 (27)
3–20%	51 (49.0)
>20% (Range 20–70%)	14 (14)
Unknown	11(11)
**Prior Lines of Systemic Treatment, No. (%) (including Somatostatin Analogs)**	
≤2	57 (55)
3	24 (23)
≥4	18 (17)
Prior Everolimus	31 (30)
Prior Somatostatin Analogs (SSA)	82 (79)
**Prior Temozolomide alone or with Capecitabine, No. (%)**	40 (38)
**Prior Liver Directed Therapy, No. (%)**	
No	57 (55)
Yes	42 (40)
Trans-arterial embolization/chemo-embolization	34 (33)
Y-90 radio-embolization	8 (8)
Radiofrequency Ablation	12(12)

Forty percent had received prior liver-directed treatment. Seventy-four percent of patients (77/104) completed all four doses, while 16 patients (15%, 16/104) discontinued treatment after two or three doses and 12 patients discontinued treatment after one dose (12%, 12/104). No patients received more than 4 doses. Among patients who discontinued treatment (n = 28), the reasons for discontinuation were treatment-related toxicities (11%, or 11/104), progressive disease (PD) (12%, or 12/104), and patient/physician preference unrelated to toxicities (5%, or 5/104).

All patients who received at least one dose of ^177^Lu-dotatate were eligible for analysis of adverse events (outlined in Table 2). Most treatment-related AEs were of grade 1 or 2 and included fatigue, nausea/vomiting, anorexia, abdominal pain, transaminase elevation, and cytopenia. Grade 3–5 AEs were seen in 39% of patients.

**Table 2:** Adverse events during and after treatment^a^.

Adverse Event	N (%)
**Any grade ≥ 1 adverse event, No. (%)**	
Elevated GFR	41 (39)
Increased ALT	33 (32)
Increased AST	45 (43)
Increased Total Bilirubin	19 (18)
Reduced Albumin	28 (27)
Anemia	75 (72)
Thromobocytopenia	69 (66)
Neutropenia	20 (19)
Fatigue	90 (87)
Nausea	67 (64)
Vomiting	32 (31)
Anorexia	41 (39)
Diarrhea	44 (42)
Constipation	41 (39)
Peripheral Neuropathy	35 (34)
Depression	39 (38)
Mucositis	10 (10)
Shortness of Breath	48 (46)
Pain	65 (63)
Local Edema	35 (34)
Rash	25 (24)
**Any grade ≥ 3 adverse event**	40 (39)
Elevated GFR	3 (3)
Increased ALT	4 (4)
Increased AST	5 (5)
Increased Total Bilirubin	5 (5)
Reduced Albumin	0 (0.0)
Anemia	10 (10)
Thromobocytopenia	8 (8)
Neutropenia	5 (5)

^a^All adverse events regardless of attribution were collected up to 8 weeks after the last dose of PRRT

Hematologic AEs included thrombocytopenia in 66.33% (58.77% G1-2, 7.7% G3-4), anemia in 72.11% (62.55% G1-2 and 9.66% G3), and neutropenia 19.2% (14.44% G1-2, 4.8% G3-4). Reflecting the long-standing nature of disease and heavy pretreatment of this cohort, 39% (40/104, 38% G1-2, 1% G3) and 21.2% (22/104, all Grade 1) had baseline anemia and thrombocytopenia, respectively, providing appropriate context for the hematologic toxicity observed. Compared to baseline, 47.1%, 19.2%, and 56.7% of patients experienced at least one grade-level worsening of anemia, neutropenia, and thrombocytopenia, respectively.

Ten percent of patients (11/104) discontinued treatment due to treatment-related adverse events (TRAEs). The reasons included cytopenia (n = 2), bowel toxicity (n = 6), acute kidney injury (n = 2), and severe fatigue (n = 1). Two patients developed severe cytopenia, leading to treatment discontinuation after the third dose. The first patient developed a hemoglobin of 6.7 g/dL and thrombocytopenia to 60,000/uL within two months of the last dose of PRRT, while the second patient developed prolonged thrombocytopenia with a low point of 44,000/uL, occurring around 7 months after the last treatment dose.

Forty percent of patients had prior exposure to temozolomide alone or in combination with capecitabine. No association was observed between prior exposure to or total cumulative dose of capecitabine/temozolomide and incidence of grade 2 or higher hematologic AEs. One patient with prior exposure to capecitabine/temozolomide and preexisting cytopenia with abnormal cytogenetics was subsequently diagnosed with myelodysplastic syndrome (MDS) 15 months after the first dose of PRRT.

GEP-NETs have a predilection to form highly vascular mesenteric masses with significant surrounding desmoplastic response. Thirty-two percent (33/104) of patients had evidence of a mesenteric mass or peritoneal involvement at baseline. In 12 of these patients (36%, 12/33), the mesenteric mass was associated with a desmoplastic reaction; tethering of adjacent bowel wall; or encasement/narrowing of, or mass-effect on, the mesenteric vessels. Overall, 7 patients (21%; 7/33) developed bowel toxicities resulting in death in two of these cases. The first patient had preexisting symptoms of mesenteric ischemia and then developed bowel ischemia requiring total parenteral nutrition (TPN) two weeks after the first dose of PRRT. He ultimately died nine weeks later.

The second patient had peritoneal carcinomatosis and subsequently developed gastric outlet obstruction three weeks after the first ^177^Lu-dotatate treatment. TPN was initiated and death occurred eight weeks later. One patient developed bowel rupture during a colonoscopy that was performed to investigate abdominal cramping and a change in stool caliber three weeks after the second dose of ^177^Lu-dotatate. This was deemed possibly related to treatment and further doses of ^177^Lu-dotatate were canceled. There were four other patients with small bowel obstructions who recovered with symptomatic management; no further doses were administered in these patients.

Reduced GFR was noted in 40% of patients (37% G1/G2, 3% G3-4). Two patients with >G3 reduction in GFR did not receive any further doses of treatment. Two patients with normal kidney function at baseline went on to develop nephrotic syndrome secondary to minimal change disease on long-term follow-up. The first patient developed AKI and nephrotic syndrome 14 months after the fourth dose of ^177^Lu-dotatate. A kidney biopsy showed prominent podocyte foot process effacement supporting minimal change disease along with IgA deposits, global glomerulosclerosis, hyperfiltration injury, and widespread nephron loss. This patient developed worsening performance status, became dialysis dependent, and died four months later. The second patient (who was on long-term lithium for history of bipolar disease) developed acute kidney injury and proteinuria 27 months after the last (fourth) dose of ^177^Lu-dotatate. Kidney biopsy showed minimal change disease and Rituximab was initiated with marked improvement in the renal function that subsequently returned to baseline.

Non-hematologic laboratory AEs included liver transaminase elevation in 43% (38% G1-2, 5% G3-4), hyperbilirubinemia in 19% (14% G1-2, 5% G3-4), and hypoalbuminemia in 27% (27% G1-2). Compared to baseline, the percentages of patients with worsening grades of AST, ALT, total bilirubin, and albumin were 27.9%, 20.2%, 17.3%, and 24%, respectively. The AE profile is summarized in Table 2.

Six patients with pancreatic NET developed acute liver failure due to rapid tumor progression during treatment that necessitated treatment discontinuation. These patients had been treated with a median of two prior lines of treatment. Five of these patients had pancreatic NET with bulky liver metastatic tumor burden encompassing ≥50% of the liver parenchyma and Ki-67 ranging between 11% to 30%. They experienced terminal liver failure within six months of starting treatment. The sixth patient also had pancreatic NET and a liver tumor burden <50%, but with liver tumor sizes of 6 cm and a Ki-67 of 40%. This patient developed terminal liver failure eight months after beginning PRRT due to liver tumor progression. The median survival for patients who developed liver failure was 7.3 months from the start of PRRT. Notably, there were 15 other patients with a liver tumor burden >50% who did not develop liver failure. Eight of these 15 patients had pancreatic NETs with a Ki-67 < 10%. Four of the 15 had small bowel primary tumors with Ki-67 between 5 and 30%. Three other patients had an unknown primary tumor, paraganglioma, and lung NET with Ki-67 between 7 and 10%.

**Fig 1:**
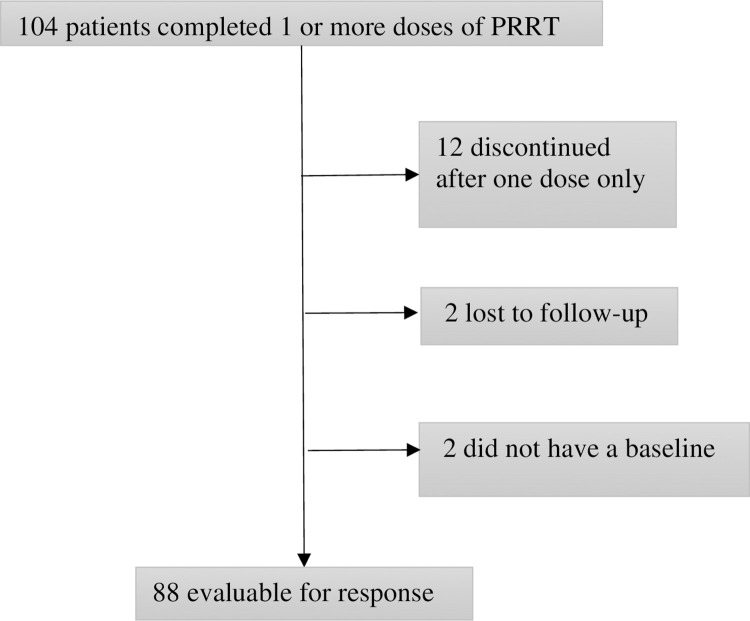
Flow diagram of patients eligible for response assessment.

Twelve patients (12%) discontinued treatment after receiving only one dose, two were lost to follow up, and two did not have a baseline scan with measurable disease for comparison. These 16 patients were excluded from the efficacy cohort (Fig 1). Thus, 88 (85%) of patients were assessable for response. The objective response rate (all partial responses) was 18% (16/88). Sixty-eight percent of the cohort (61/88) had stable disease, while 13% (11/88) had progressive disease. Median time to treatment failure (TTF) or death was 27.6 months for the entire cohort, but was significantly longer (p = 0.001) in small bowel NET (37.3 months, n = 49) when compared to pancreatic (13.2 months, n = 32) or lung NETs (15.8 months, n = 7).

**Fig 2:**
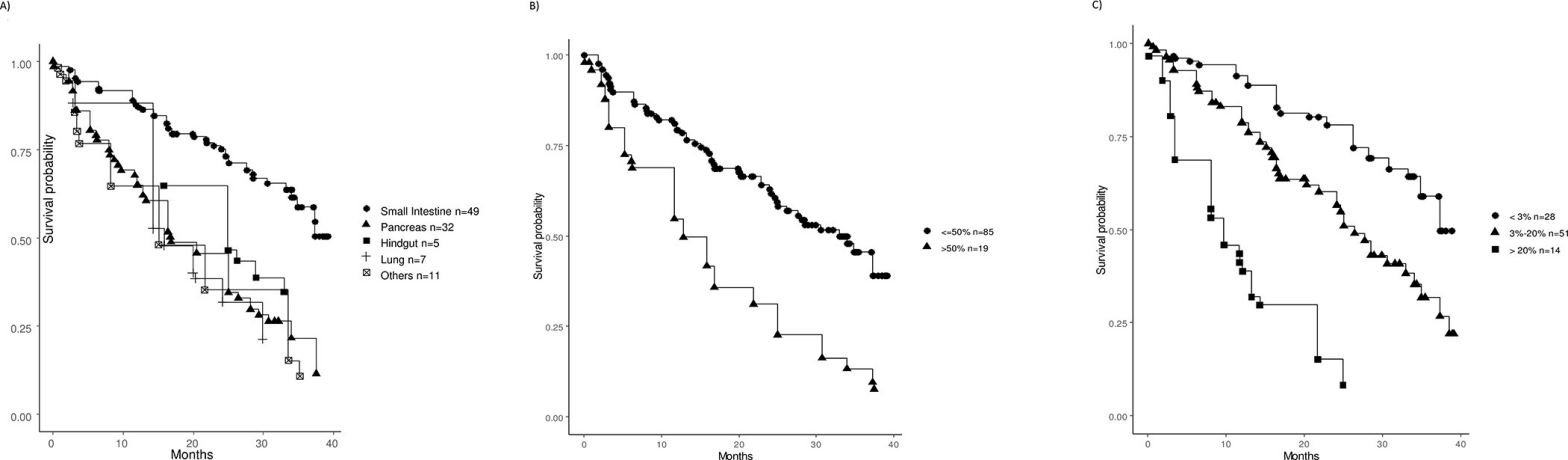
Kaplan-Meier curves of time to treatment failure by A) primary site; B) liver tumor burden; and C) Ki-67%.

Patients with a liver tumor burden of >50% (n = 20) had significantly shortened TTF (p<0.001) of 12.8 months when compared to patients with a tumor burden <50% (30.5 months). TTF was also significantly associated with Ki-67 (p<0.001) with a median of 37.3 months, 25 months, and 9.7 months in grades 1–3, respectively (Fig 2).

There was no significant difference in TTF based on prior liver-directed therapy. In an age-adjusted multi-variate model, primary site, Ki-67 percentage, and liver tumor burden >50% were independently significant predictors of TTF (Table 3).

**Table 3:** Multivariable analysis of factors associated with time to treatment failure.

	HR (95% CI)	P value
**Age**	1.02 (0.99–1.04)	0.289
**Site**		
Small intestine	1 [Reference]	NA
Pancreas	2.53 (1.2–5.32)	0.014
Hindgut	2.34 (0.85–6.42)	0.099
Lung	2.64 (0.83–8.45)	0.101
Others	3.61 (1.16–11.2)	0.026
**Liver tumor burden > = 50%**	3.08 (1.59–5.98)	< .001
**Ki-67**		
<3%	1 [Reference]	NA
3–20%	2.02 (0.96–4.28)	0.066
>20%	6.5 (2.39–17.7)	< .001

## Discussion

With the approval of PRRT with ^177^Lu-dotatate, there has been widespread uptake and use of this treatment modality at tertiary centers across the US for the treatment of NETs. An in-depth analysis of the clinical efficacy, safety, and toxicity profile in the real-world setting is critical to understanding the benefits and risks to patients who may differ from research study subjects in key ways such as extensive pretreatment and very advanced nature of disease. We summarize clinical data from a large tertiary academic center of a group of patients with NET treated with ^177^Lu-dotatate. The cohort was heavily pretreated, with 40% having received three or more prior lines of systemic treatment. Forty percent had received prior transarterial embolization to the liver. We observed an objective response rate (18%) that was identical to that reported in the NETTER-1 trial. The median TTF was 27.6 months for the entire cohort and 13.2 months in pancreatic NETs. A review of patients from another tertiary center showed a progression-free survival of 21.6 months for the study group and 13.3 months for patients with pancreatic primary tumors [3]. A multi-center review of patients treated with ^177^Lu-dotatate showed an objective response rate (18.7%) that was nearly identical to our findings [12]. We therefore confirm the reproducibility of these findings, which will be useful data to help design future prospective studies in pretreated populations.

The safety profile was also consistent with that experienced by the NETTER cohort with some important differences. Only 3% of the cohort experienced >G3 increase in serum creatinine. Increases in liver transaminases and bilirubin post-PRRT were found to be common, but generally of mild severity and >G3 in only around 5% of patients. Greater than grade 3 hematologic adverse events were noted in up to 10% of patients, underscoring the importance of close monitoring of blood counts in heavily pretreated patients. Discontinuation of PRRT owing to intolerance or an adverse event occurred in 10% of patients—slightly more frequent than that reported in NETTER‐1 (6%), likely reflecting the generally advanced nature of disease.

The occurrence of bowel-related complications post-PRRT is a particularly challenging sequelae of treatment that is difficult to distinguish from the natural course of disease progression in patients with mesenteric/peritoneal tumor involvement. Twenty-one percent of patients with baseline peritoneal/mesenteric involvement developed bowel toxicities (7/33). Two patients had grade-5 events: gastric outlet obstruction and bowel ischemia. A single-institution retrospective review from 2021 of 81 patients with mesenteric/peritoneal involvement treated with PRRT showed bowel obstructions in 6% of patients including two deaths [7]. The risk of bowel toxicity may be mitigated with the use of prophylactic dexamethasone, which was not yet routinely employed in the time frame covered by our cohort. This could explain the relatively higher rates of bowel toxicity experienced when compared to the aforementioned study. It is prudent to discuss risks and benefits with patients who have preexisting peritoneal/mesenteric involvement or symptomatic mesenteric ischemia who are being considered for PRRT.

Two patients developed delayed-onset minimal-change nephrotic syndrome from 14 to 27 months after the last dose of ^177^Lu-dotatate. A slow persistent decline in the creatinine clearance of a median of 3.8% per year has been reported with ^177^Lu-dotatate PRRT though up to 5% of patients have an accelerated decline of >15% per year [13]. Further studies are needed to understand the mechanism underlying this toxicity.

We identified six patients who developed rapid tumor progression and terminal liver failure within one year (range: 3–364 days) after starting treatment with PRRT. Every one of these patients had pancreatic primaries, Ki-67 >10% along with extensive liver tumor burden (≥50%) and/or bulky disease (>5 cm). An analysis of data from patients with mid-gut NETs (NETTER-1) suggests that, within the ^177^Lu-dotatate arm, there was no significant difference in PFS amongst patients with low/moderate/high liver tumor burden. The absence of a large target lesion (defined as >30mm) however was associated with better PFS (p = 0.02) [14]. Taken together, this likely indicates that bulky liver lesions in patients with pancreatic NETs have a uniquely poor prognosis, reflecting an aggressive underlying disease biology. Overall, these observations will need to be studied prospectively in predefined stratified subsets of patients to better understand their significance.

The single-center nature and limited numbers are limitations of this study, which preclude robust subset analysis. The overall reproducibility of the semi-quantitative visual estimation of liver tumor burden is a potential limitation. Real-world experience gained from studying patients treated with ^177^Lu-dotatate are valuable as they may be broadly applicable to newer modalities of PRRT with alpha-emitting agents.

## Conclusion

Overall, ^177^Lu-dotatate was shown to be safe and effective in a population of heavily pretreated patients. We recommend caution in the subset of patients with preexisting bowel disease or mesenteric vessel involvement. An emerging signal for the potential to develop minimal change nephrotic syndrome was also identified, which will require prospective validation.

## Supporting information

S1 Checklist STROBE checklist. (DOCX)

S1 Data (DOCX)

S1 File (PDF)
