# Supplementary material for: Safety and efficacy of peptide receptor radionuclide therapy in neuroendocrine tumors: A single center experience
Source: PLoS One. 2024 May 15;19(5):e0298824. doi: 10.1371/journal.pone.0298824 (PMC11095666; doi:10.1371/journal.pone.0298824)
Supplement: S1 Data — (DOCX) [file pone.0298824.s002.docx]

Sent Date: 12/27/2018 4:33 pm

From: Ellen Patricia <patricia.1@osu.edu>

To: Bhavana Konda <konda.8@osu.edu>

Cc: April Green [green.1655@osu.edu](mailto:green.1655@osu.edu),Matthew Brown [Matthew.Brown@osumc.edu](mailto:Matthew.Brown@osumc.edu)

12/27/2018

Study Number: 2018C0187
Study Title: OSU-18283: A Retrospective Analysis of the Outcomes of Patients with Rare Neuroendocrine tumors treated with Various therapies at the Ohio State University Comprehensive Cancer Center

Type of Review: Initial Submission

Review Method: Expedited

Date of IRB Approval: 12/27/2018
Date of IRB Approval Expiration: 12/27/2019

Expedited category: #5

Dear Bhavana Konda,

The Ohio State Cancer IRB **APPROVED** the above referenced research.

In addition, the following were also approved for this study:

- Children
- Waiver of Assent
- Waiver of Parental Permission
- Waiver of Consent Process
- Full Waiver of HIPAA Research Authorization

As Principal Investigator, you are responsible for ensuring that all individuals assisting in the conduct of the study are informed of their obligations for following the IRB-approved protocol and applicable regulations, laws, and policies, including the obligation to report any problems or potential noncompliance with the requirements or determinations of the IRB. Changes to the research (e.g., recruitment procedures, advertisements, enrollment numbers, etc.) or informed consent process must be approved by the IRB before implemented, except where necessary to eliminate apparent immediate hazards to subjects.

This approval is issued under The Ohio State University's OHRP Federalwide Assurance #00006378 and is valid until the expiration date listed above. ***Without further review, IRB approval will no longer be in effect on the expiration date.*** To continue the study, a continuing review application must be approved before the expiration date to avoid a lapse in IRB approval and the need to stop all research activities. A final study report must be provided to the IRB once all research activities involving human subjects have ended.

Records relating to the research (including signed consent forms) must be retained and available for audit for at least 5 years after the study is closed. For more information, see university policies, [Institutional Data](https://urldefense.com/v3/__https:/ocio.osu.edu/sites/default/files/assets/Policies/InstitutionalData.pdf__;!!AU3bcTlGKuA!Hi1IBrjgQbBWk8vYJ_H2pUl7B3cd1CammpY71ZMfcWehYyPo6Ocwan8id-EZ37CA1WtAtsyQEQWgQsnjWGEDhJPO-iBM$) and [Research Data](https://urldefense.com/v3/__http:/orc.osu.edu/files/ResearchDataPolicy.pdf__;!!AU3bcTlGKuA!Hi1IBrjgQbBWk8vYJ_H2pUl7B3cd1CammpY71ZMfcWehYyPo6Ocwan8id-EZ37CA1WtAtsyQEQWgQsnjWGEDhIHL6bEX$).

Human research protection program policies, procedures, and guidance can be found on the [ORRP website](https://urldefense.com/v3/__http:/orrp.osu.edu/__;!!AU3bcTlGKuA!Hi1IBrjgQbBWk8vYJ_H2pUl7B3cd1CammpY71ZMfcWehYyPo6Ocwan8id-EZ37CA1WtAtsyQEQWgQsnjWGEDhF1Z-QkL$).

William Carson, III, MD, Chair
Ohio State Cancer IRB
