# Supplementary material for: Safety and efficacy of peptide receptor radionuclide therapy in neuroendocrine tumors: A single center experience
Source: PLoS One. 2024 May 15;19(5):e0298824. doi: 10.1371/journal.pone.0298824 (PMC11095666; doi:10.1371/journal.pone.0298824)
Supplement: S1 File — (PDF) [file pone.0298824.s003.pdf]

## Felix, Josephine

| | |
|---|---|
| **From:** | Buck-IRB <irbinfo@osu.edu> |
| **Sent:** | Thursday, September 8, 2022 4:44 PM |
| **To:** | Konda, Bhavana |
| **Cc:** | nelson.1845@osu.edu; felix.95@osu.edu |
| **Subject:** | Annual Status Report Confirmed for #2018C0187 |
| | |
| **Follow Up Flag:** | Follow up |
| **Flag Status:** | Completed |
| | |
| **Categories:** | 1. Continuing Reviews |

THE OHIO STATE UNIVERSITY

**Office of Responsible Research Practices**
300 Research Administration building
1960 Kenny Road
Columbus, OH 43210-1063

orrp.osu.edu

09/08/2022

Study Number: 2018C0187
Study Title: OSU-18283: A Retrospective Analysis of the Outcomes of Patients with Rare Neuroendocrine tumors treated with Various therapies at the Ohio State University Comprehensive Cancer Center

Type of Review: Annual Status Report

Date of Annual Status Report Confirmation: 09/08/2022
Date of IRB Approval Expiration: 09/08/2023

Dear Bhavana Konda,

The Office of Responsible Research Practices has **CONFIRMED CONTINUATION** of the above referenced research.

As Principal Investigator, you are responsible for ensuring that all individuals assisting in the conduct of the study are informed of their obligations for following the IRB-approved protocol and applicable regulations, laws, and policies, including the obligation to report any problems or potential noncompliance with the requirements or determinations of the IRB. Changes to the research (e.g., recruitment procedures, advertisements, enrollment numbers, etc.) or informed consent process must be approved by the IRB before implemented, except where necessary to eliminate apparent immediate hazards to subjects.

This confirmation is issued under The Ohio State University's OHRP Federalwide Assurance #00006378 and is valid until the expiration date listed above. ***Without further review, IRB approval will no longer be in effect on the expiration date.*** To continue the study, an annual status report or continuing review application must be approved before the expiration date to avoid a lapse in approval and the need to stop all research

activities. A final study report must be provided to the IRB once all research activities involving human subjects have ended.

Records relating to the research (including signed consent forms) must be retained and available for audit for at least 5 years after the study is closed. For more information, see university policies, Institutional Data and Research Data.

Human research protection program policies, procedures, and guidance can be found on the ORRP website.
